# New Appliances and Things Medical

**Published:** 1898-09-03

**Authors:** 


### NEW APPLIANCES AND THINGS MEDICAL.

[We shall be glad to receive, at our Office, 28 & 29, Southampton Street, Strand, London, W.C., from the manufacturers, specimens of all new preparations and appliances which may be brought out from time to time.]

### MINERAL WATERS.—ULRICUS AND BELVEDRA.

("Passug," 39, Eastcheap, London, E.C.)

We have received samples of the above waters, which are imported from the Gorge of the Rabinsa, near Coire, Switzerland. The former is a strongly alkaline water, containing traces of iron and bromine. It contains a considerable quantity of free carbonic acid, and hence is slightly effervescent on opening. It is specially recommended for diabetic cases, and from a consideration of its ingredients we should be inclined to think that a course of them would be decidedly beneficial for patients suffering from this condition. The Belvedra is a first-class ferruginous table water, and, for an iron-containing water, not noticeably disagreeable or astringent. For cases in which iron in an alkaline form is indicated, this water will be found of value.

### POMONA CIDER.

(John Watkins, Pomona Farm, Withington, Hereford.)

This bottled cider, which has deservedly taken a large number of prizes, has most favourably impressed us by reason of its uniform excellence and agreeableness of flavour. The proprietor of this brand exercises personal supervision, not only in the manufacture of the cider but in the selection of the apples from which the juice is expressed. The works are situated in the very heart of the great apple-growing district in Herefordshire, and, although the fruit is bought in immense quantities from neighbouring farmers, the proprietor claims never to buy cider in an unrefined condition. The work of manufacture is conducted entirely under his own direction on the most modern and approved principles. Many most enlightened physicians are now ordering cider for their gouty and rheumatic patients, and, had a reliable and pure cider been within reasonable reach of the public, its usefulness in such conditions would have been established as a popular beverage at a much earlier date. Badly fermented cider, and cider with traces of lead, was responsible in the past for the bad odour in which it was for a long time held by the medical profession. With such a cider as the "Pomona brand" these reproaches are entirely unjustifiable, and when its merits are more widely known it will doubtless do much to dissipate any remaining prejudice that may exist in the minds of many persons regarding ciders generally. For our part, we have long recommended cider in moderation for consumption among the rheumatic and gouty; its alcoholic percentage is so low that it may almost rank among teetotal beverages. It has, however, an exhilarating effect, and a slightly stimulating influence on kidneys and liver. We recommend the Pomona brand strongly to the unconverted as well as to those who are familiar with the qualities of a good cider.

### VIN URANE (PESQUI).

(Fassett and Johnson, 31, Snow Hill, London, E.C.)

This is a wine which, according to the analysis supplied, contains Médoc, nitrate of uranium, bromide of lithium, pepsine, quinine, glycerine, &c. These constituents may be relied upon to effect two specific ends. (1) An improvement in the general condition, owing to the tonic and digestive qualities of the wine, pepsine, quinine, &c. (2) An improvement in special condition owing to the effect of the uranium nitrate, which according to some authorities has a markedly favourable influence on the formation of sugar. It should, however, be given under medical advice. It cannot be regarded as a "cure." A cure for diabetes has yet to be discovered, and we cannot recommend every man to become his own diagnostician and prescribe himself "Vin Urané" when he observes the following symptoms: Thirst, depression, lassitude, irritability without apparent cause, diminution of both physical and mental faculties. These are the symptoms of the dipsomaniac and the hypochondriac, and for either of these Vin Urané would prove a most insidious and dangerous remedy. For the diabetic we are prepared to recommend a cautious trial of this medicated wine.

